# Targeting and Function of the Mitochondrial Fission Factor GDAP1 Are Dependent on Its Tail-Anchor

**DOI:** 10.1371/journal.pone.0005160

**Published:** 2009-04-02

**Authors:** Konstanze M. Wagner, Marcel Rüegg, Axel Niemann, Ueli Suter

**Affiliations:** Institute of Cell Biology, Department of Biology, ETH Zürich, Zürich, Switzerland; National Institutes of Health, United States of America

## Abstract

Proteins controlling mitochondrial dynamics are often targeted to and anchored into the mitochondrial outer membrane (MOM) by their carboxyl-terminal tail-anchor domain (TA). However, it is not known whether the TA modulates protein function. GDAP1 is a mitochondrial fission factor with two neighboring hydrophobic domains each flanked by basic amino acids (aa). Here we define GDAP1 as TA MOM protein. GDAP1 carries a single transmembrane domain (TMD) that is, together with the adjacent basic aa, critical for MOM targeting. The flanking N-terminal region containing the other hydrophobic domain is located in the cytoplasm. TMD sequence, length, and high hydrophobicity do not influence GDAP1 fission function if MOM targeting is maintained. The basic aa bordering the TMD in the cytoplasm, however, are required for both targeting of GDAP1 as part of the TA and GDAP1-mediated fission. Thus, this GDAP1 region contains critical overlapping motifs defining intracellular targeting by the TA concomitant with functional aspects.

## Introduction

Mitochondria are dynamic organelles that constantly fuse and fragment. Critical components that regulate and coordinate fusion and fission of the mitochondrial inner and outer membrane (MOM) have been identified [1], and mutations in genes of fusion and fission factors have been linked to neurodegenerative diseases and prenatal lethality [2], [3].

Many proteins involved in the regulation of mitochondrial dynamics are located at the MOM and contain a C-terminal membrane tail anchor (TA). For example, mitofusins span the MOM twice with the N- and the C-terminus facing the cytosol [4]. A specific class of TA proteins, however, termed here “classical” TA proteins, have a cytosolic N-terminal part that is membrane-embedded via a single hydrophobic transmembrane segment close (less than thirty amino acids (aa)) to the C-terminus. This TA-domain is sufficient for efficient posttranslational targeting and integrates into the membrane [5], [6], [7].

Notable examples of classical TA-proteins of the MOM include small import receptors of the preprotein translocase of the outer mitochondrial membrane (TOM complex) [8]. Furthermore various proteins involved in mitochondrial dynamics belong to the group of TA proteins (Table 1) including the mitochondrial fission factors Fis1 [9] and Mff [10], and the proapoptotic protein Bak which also regulates mitochondrial morphology in non-apoptotic conditions [11].

**Table 1:** Characteristics of TMDs of selected MOM TA proteins and mutants thereof.

Protein	TMD sequence	Residues in TMD	Hydrophobicity	
			Total	Mean
Vamp1B	**…K** MMIMLGAICAIIVVVIVS **K…**	18	45,7	**2,54**
Bak	**…R** *DP* ILTVMVIFGVVLLGQFVV **H…**	18	40,1	**2,23**
Gdap1 TMD	**…K** VLGSTLVVGLLVGMGYFAFMLF **R…**	22	49,0	**2,23**
Bcl-X	**…R** WFLTGMTVAGVVLLGSLFS **R…**	19	39,9	**2,10**
Fis1	**…K** *D* GLVGMAIVGGMALGVAGLAGLIG *LAVS* **R…**	23	46,4	**2,02**
Tom20	**…R** *NS* AIAAGVCGALFIGYCIYF **D…**	18	34,1	**1,89**
Mff	**…E** MVMYSITVAFWLLNSWLWF **R…**	19	35,1	**1,85**
Bcl-2	**…K** TLLSLALVGACITLGAYLG **H…**	19	34,6	**1,82**
Omb	**…K** *SC* WAYWILPIIGAVLLGFLY **R…**	18	30,4	**1,69**
Tom5	**…K** QAAYVAAFLWVSPMIWHLV **K…**	19	26,4	**1,39**
Mfn-1 TMD2*	**…K** LISVTSSMYGALYLY *E* **R…**	15	20,8	**1,39**
Miro-2	**…R** GLLGVVGAAVAAVLSFSLYRVLV **K…**	23	30,9	**1,34**
Gdap1 HD1	**…K** VLGHVNNILISAVLPTAF **R…**	18	19,3	**1,07**
Mfn-1 TMD1*	**…L** TSRTSMGIIVVGGVIW **K…**	16	17,0	**1,06**
Miro-1	**…K** *SSTF* WLRASFGATVFAVLGFAMYKALL **K…**	23	22,8	**0,99**
GDAP1hy	**…K** VLGSTaVVGLLVGsGYaAyMLF **R…**	22	38,5	**1,75**
TMDscr	**…K** VGMALFVTGMSYFGLLVGLLVF **R…**	22	49,0	**2,23**

Total hydrophobicity: Sum of values of TMD between basic aa (in bold), according to GES hydrophobicity scale [28]. Italics indicate aa between flanking basic aa and the longest hydrophobic stretch (not included in the calculation). Lower case letters indicate mutated aa. *protein is not a classical TA protein.

Although TA-proteins lack a N-terminal targeting signal, they specifically integrate into a limited number of organelles such as the endoplasmic reticulum (ER), mitochondria, peroxisomes, and chloroplasts [9]. The best-studied ER- and MOM-targeted TA proteins share structural and mechanistic features but strongly differ in other aspects. Specificity of targeting of classical TA proteins to the MOM is mediated by basic aa flanking a short TMD while in ER-targeted proteins the TMD is in general longer and flanked by neutral or acidic aa [5], [6], [7], [12], [13]. ER-targeted TA proteins with more hydrophobic TMDs require accessory factors for translocation across a lipid bilayer while TMDs with limited hydrophobicity can translocate without assistance [14]. Chaperones and a targeting factor for assisted TA protein ER integration have been identified [15], [16]. The mechanism of TA-MOM protein integration remains a matter of some debate. Studies suggest that Tom40 is an essential component during the integration process of classical TA proteins [9] but Setoguchi et al. [17] found that MOM-targeted TA proteins even with a more hydrophobic TMD are still capable of unassisted translocation. Despite progress in understanding the underlying cellular machinery of TA protein targeting and insertion, the functional role of the tail region remains elusive [5], [9]. The exchange of the TA of Fis1 with the TA of Tom5 or Tom6 demonstrated that the TA is required for targeting of Fis1 but not for its activity in yeast. Yet, these overexpressed fusion proteins were partially integrated into the TOM complex, suggesting that the TA of Tom5 and Tom6 might additionally act as an assembly signal [18]. Further clarification of the important issue whether the TA of classical TA proteins is, in addition to its function in membrane anchoring, of other functional significance, requires appropriate assays that assess protein activity in response to alterations of the TA.

The nervous system-enriched GDAP1 (ganglioside induced differentiation associated protein 1) is a mitochondrial fission factor located at the MOM [19]. Mutations in *GDAP1* lead to the peripheral neuropathy Charcot-Marie-Tooth disease (CMT), affecting both Schwann cells, the myelinating glia of the peripheral nervous system, and neurons [2]. GDAP1 is structurally related to cytosolic glutathione *S*-transferases (GSTs) [20] but GST activity of bacterially-expressed recombinant protein has not been found [21], [22, our unpublished data]. Similar to mitofusins, GDAP1 contains two hydrophobic stretches at the C-terminus and is an integral MOM protein with the more C-terminal hydrophobic domain sufficient for mitochondrial targeting [19].

This study was aimed at determining the precise topology of GDAP1, the motifs involved in MOM targeting and integration, and the relationship between TA structure and the fission function of the protein.

## Results

### GDAP1 is a tail-anchored protein with a single transmembrane domain

**Figure 1:**
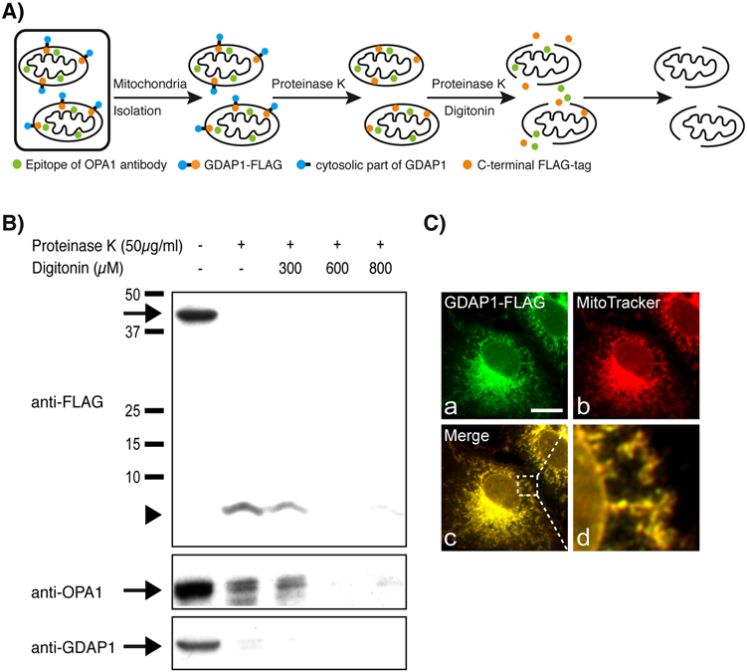
GDAP1 membrane topology. (A) Graphic of experimental strategy. (B) Western blot of mitochondria-enriched fractions derived from GDAP1-FLAG-transfected HeLa cells treated with proteinase K for 30 minutes at 4°C with increasing digitonin concentrations. Without digitonin, the cytosolic N-terminal part of GDAP1-FLAG is degraded (arrow) and only the C-terminal 5 kDa fragment is detected (arrowhead). After membrane permeabilization, the C-terminal fragment is gradually degraded confirming its location in the IMS. The IMS protein Opa1 served as control. (C) Confocal immunofluorescence microscopy of transfected COS-7 cells expressing GDAP1-FLAG (a–d). Co-stainings: MitoTracker (mitochondria). Bars, 10 µm.

To determine the topology of GDAP1, we required an appropriate epitope-tagged version of the protein. Large C-terminal tags severely impair the posttranslational translocation of ER-targeted TA proteins [14]. Similarly, we found that C-terminal tags that significantly extend GDAP1, like EGFP, cause extensive mitochondrial aggregation and a significant loss of mitochondrial localization compared to untagged GDAP1. Such EGFP-tagged proteins also attach only peripherally to mitochondria (Suppl. Figs. S1, S4). To avoid these artifacts, we exchanged eight C-terminal aa of GDAP1 against the short FLAG-tag (DYKDDDDK) to maintain the length of the C-terminus (Fig. 1C). HeLa cells were transfected with the GDAP1-FLAG construct and mitochondria isolated after 24 hours, digested with protease K in the presence or absence of the detergent digitonin, followed by Western blot analysis (Fig. 1A) [23]. Without detergent, the anti-Flag antibody detected a proteinase K-resistant fragment of 5 kDa, consistent with the expected molecular weight of a peptide containing the C-terminus and the more C-terminally located hydrophobic segment of GDAP1 (Fig. 1B, arrowhead). This fragment and a control protein located in the intermembrane space (IMS), OPA1, were digested upon membrane solubilization by increasing digitonin concentrations. Comparable results were obtained with a C-terminally FLAG-tagged full-length GDAP1 (data not shown). Together with our previous results demonstrating that the more C-terminally located hydrophobic domain and its immediate flanking regions are sufficient for GDAP1 targeting to mitochondria [19], these findings establish that GDAP1 integrates into the MOM as a classical TA protein with a single transmembrane domain and the C-terminus located in the IMS. Consequently, we have renamed the most C-terminal hydrophobic segment of GDAP1 to transmembrane domain (TMD) and the other hydrophobic portion to hydrophobic domain 1 (HD1).

### The classical tail-anchored proteins GDAP1 and Fis1 but not Mfn2 integrate into isolated mitochondria-enriched membrane preparations

**Figure 2:**
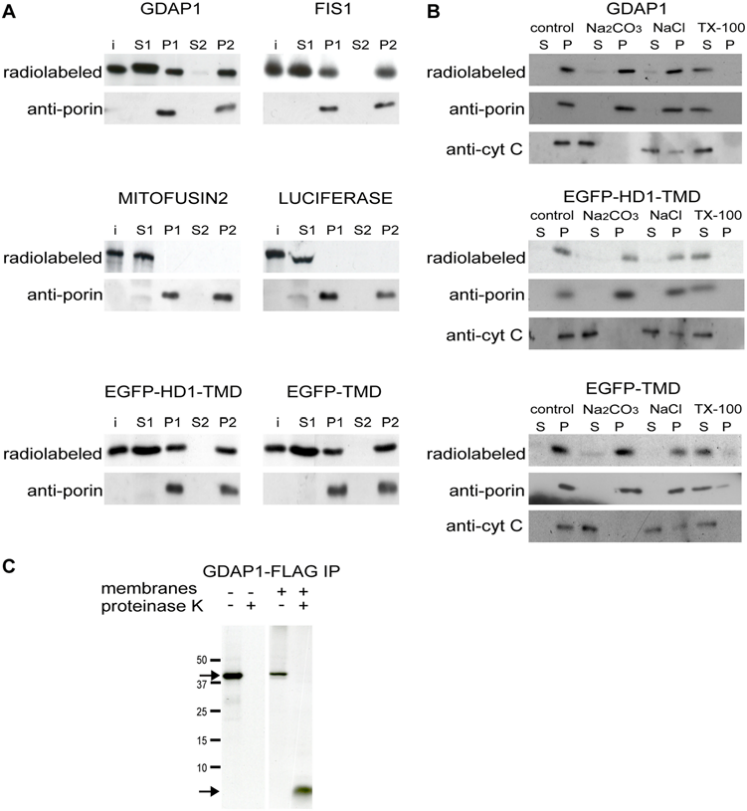
Integration assay of in vitro-synthesized TA-proteins. (A) The post-nuclear supernatant of HeLa cells was incubated with the *in vitro*-translates of GDAP1, Fis1, Mfn2, Luciferase, EGFP-HD1-TMD or EGFP-TMD. Mitochondria were enriched in a differential centrifugation approach (S1, P1, S2, P2). GDAP1, Fis1, EGFP-HD1-TMD and EGFP-TMD showed co-sedimentation with the mitochondrial marker porin. Mfn2 and Luciferase remained in the supernatant. (B) The post-nuclear supernatant of HeLa cells was incubated with the *in vitro*-translated GDAP1, Fis1, EGFP-HD1-TMD and EGFP-TMD and the mitochondrial pellet was resuspended in buffer (control), in 1 M NaCl, 0.1 M carbonate (pH 11), or in buffer with 0.1% TritonX-100, and centrifuged to separate the soluble protein supernatants (S) from membranous pellets (P). All *in vitro*-translated proteins and the integral membrane protein porin could be sedimented and became soluble only upon treatment with detergent. Unlike the integral membrane proteins cytochrome C can only be sedimented under control conditions. (C) Immunoprecipitation of *in vitro* translated GDAP1-FLAG with an anti-FLAG antibody without and after membrane integration and with or without proteinase K (50 mg/ml) digest. The upper arrow points to the undigested immuno-precipitated full length GDAP1-FLAG. The lower arrows indicates the 5 kD fragment still detected after membrane integration and protease digest.

**Figure 3:**
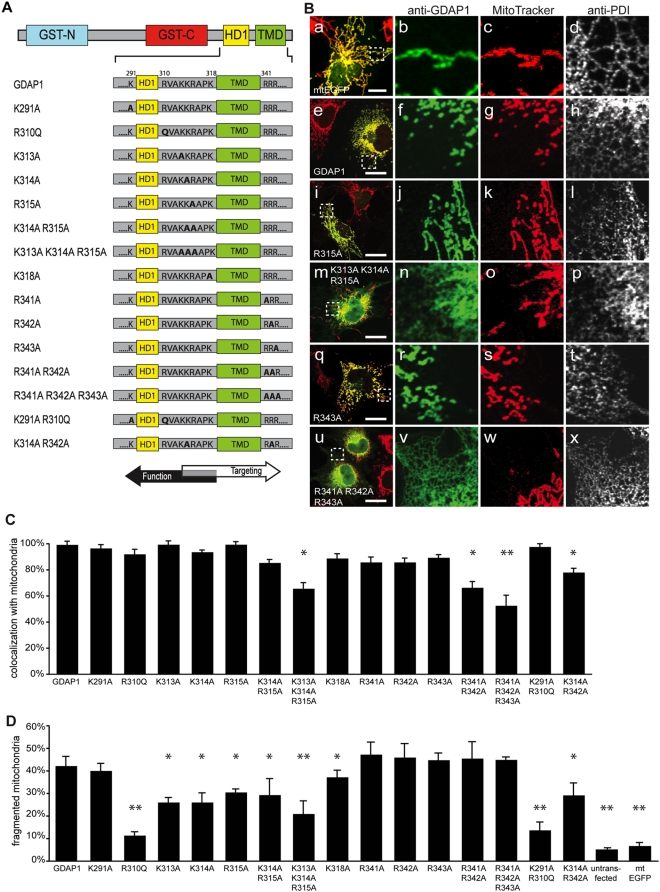
TA-associated critical basic residues for GDAP1 localization and function. (A) Schema of GDAP1 and aa sequence of C-terminal part of wt GDAP1 (GDAP1) and mutants (bold letters: altered aa sequence). (B) Confocal immunofluorescence microscopy of transfected COS-7 cells expressing mtEGFP as control (a–d) wt GDAP1 (e–h) and mutant proteins (i–x). Co-stainings: MitoTracker (mitochondria), PDI (ER). Bars, 10 µm. (C) Quantification of GDAP1 and mutants colocalized with MitoTracker. (D) Quantification of mitochondrial morphology.

To test whether GDAP1 can integrate into membranes like a classical TA-protein, we established an integration assay similar to assays published by Henderson et al. [24] and Setoguchi et al. [17]. We added radiolabeled *in vitro* translated GDAP1, Fis1, Mfn2 or luciferase to the post-nuclear supernatant of Hela cells (Fig. 2A). After an hour of incubation on ice, the soluble fraction (S1) and a mitochondrial-enriched fraction (P1) were separated by centrifugation. Mfn2 and luciferase remained in S1. In contrast, GDAP1 and Fis1 co-sedimented with the mitochondrial marker porin in two serial centrifugation steps (P1, P2). Radiolabeled GDAP1 and Fis1 integrated with an efficiency of 25±2% and 28±2% (s.d.), respectively, under the given conditions and remained membrane-bound after the second centrifugation step (P2). To evaluate the role of the cytosol-exposed region of GDAP1 on the integration, we used EGFP fused to HD1 and TMD together (EGFP-HD1-TMD; aa 291–358), or the TMD alone (EGFP-TMD; aa 311–358). Both constructs contain the flanking basic aa and the TMD C-terminal aa (Fig. 3). Comparable to full-length GDAP1, the chimeric proteins integrated also efficiently (25%±3%) and co-sedimented with porin. Hence, the TMD (including its flanking basic aa and the C-terminus) is sufficient for membrane integration. To rule out that GDAP1 is only peripherally associated with membranes, we repeated the original integration assay but treated P1 with 1 M sodium chloride or 0.1 M carbonate buffer (pH 11) [19]. Consistent with membrane integration, GDAP1 could be sedimented again in the second centrifugation step. GDAP1 was only released into the supernatant upon treatment with detergent, comparable to the integral mitochondrial membrane protein porin, while cytochrome C was released into the supernatant upon treatment with carbonate or high salt. EGFP-HD1-TMD and EGFP-TMD behaved identical to the GDAP1 full-length protein in this assay (Fig. 2B). The GDAP1-EGFP fusion protein can be co-sedimented with the mitochondrial-enriched fractions P1 and P2, but is released into the supernatant upon treatment with high salt or carbonate (Suppl. Fig. S2). This supports the previous observation (Suppl. Fig. S1) that the C-terminal EGFP-fusion interferes with the integration into the MOM. Finally, to confirm the GDAP1 membrane integration in our in vitro assay, we performed a proteinase K digest of membrane-integrated radiolabeled GDAP1-FLAG. Upon immuno-precipitation with the anti-FLAG antibody the full-length GDAP1-FLAG was detected in the control reaction without protease. In the presence of proteinase K we precipitated a proteinase K-resistant fragment of 5 kDa (Fig. 2C). This fragment represents the membrane-protected C-terminal region of GDAP1-FLAG confirming the proper integration of in vitro translated GDAP1-FLAG into membranes comparable to the integration shown in Figure 1. Without membrane integration GDAP1-FLAG is degraded by proteinase K and no proteinase resistant fragment was precipitated (Fig. 2C).

Taken together, these results support the suggested topology of GDAP1 in the MOM as a classical TA-protein with a single TMD. This is backed by the following additional evidence: First, GDAP1 integrated in membranes like a classical TA-protein comparable to Fis1 in our *in vitro* assay; Second, in contrast, the two TMD-containing MOM protein Mfn2 did not integrate; Third, the TMD of GDAP1 was sufficient to mediate integration.

### Positively charged amino acids surrounding the TMD determine GDAP1 mitochondrial targeting and function

Basic aa flanking the TMD are essential for MOM targeting of classical TA proteins, whereas neutral or acidic residues at the C-terminus of TA proteins lead to a predominantly ER localization [5], [6], [12]. Since both hydrophobic domains of GDAP1 are flanked by basic aa, we examined their effect on mitochondrial localization by systematic exchanges to neutral aa, either singly or in combination (Fig. 3A). Subcellular localization was analyzed in transiently transfected COS-7 cells using MitoTracker and anti-PDI (for ER) as organelle markers on single plane confocal images. MtEGFP control-transfected and Wildtype (wt) GDAP1 transfected COS-7 cells showed a typical mitochondrial pattern (Fig. 3B, a–h). ER localization distinguishable from mitochondrial staining, especially focusing on the periphery of cells, was not detected. Single exchanges of basic aa either N- or C-terminal of the TMD did not alter the strict mitochondrial localization (Fig. 3B, i–l; q–t, 3C), nor did the exchange of two basic aa N-terminal of the TMD (GDAP1_[K314A R315A]_, Fig. 3C) or flanking HD1 (GDAP1_[K291A R310Q]_, Fig. 3C). Only alteration of three basic aa N-terminal of the TMD led to mistargeting to the ER (GDAP1_[K313A K314A R315A]_, Fig. 3B m–p; 3C). In contrast, the exchange of two residues C-terminal or flanking the TMD (GDAP1_[R341A R342A]_; GDAP1_[K314A R342A]_) already led to significant loss of mitochondrial localization (Fig. 3C). This effect was even more pronounced in the triple mutant GDAP1_[R341A R342A R343A]_ C-terminal of the TMD (Fig. 3B u–x; 3C).

We also observed that mitochondrial morphology was altered compared to wt GDAP1 when some mutants were expressed (Fig. 3B, D). This was not due to different expression levels as determined by Western blots (Suppl. Fig. S5a). Quantification revealed that all mutant proteins with aa exchanges between HD1 and the TMD showed a significant reduction in fission activity compared to wt GDAP1, shifting the morphology to more tubular and aggregated mitochondria (Fig. 3B i–p). Hence, the mutated aa are critical for both correct targeting and GDAP1-mediated fission. In contrast, variants with single aa exchanges C-terminal of the TMD revealed no alteration in mitochondrial morphology and targeting (Fig. 3D).

Taken together, (1) Mitochondrial targeting of GDAP1 is not critically dependent on individual basic aa within the TA; (2) C-terminally TMD-flanking clusters of basic aa play a key role in targeting; (3) A cluster of basic aa N-terminally bordering the TMD is also required for mitochondrial localization; strikingly, even individual aa of this cluster are concurrently crucial for GDAP1 function; (4) The residues immediately adjoining HD1 N- or C-terminally (K291, R310) are not required for targeting, but R310 is required for fission. Interestingly the mutation R310Q was found in patients with CMT [19].

### The correctly ordered amino acid sequence of HD1, but not of the TMD, is critical for mitochondrial fission activity

**Figure 4:**
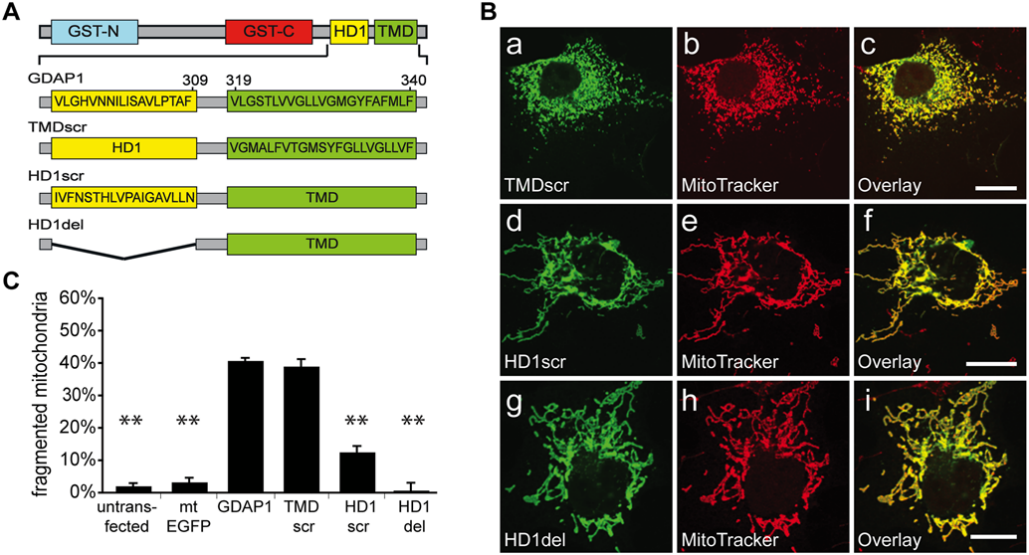
Role of TMD and HD1 amino acid sequences in GDAP1-mediated fission. (A) Schematic representation of the aa sequence of the TMD and HD1 of wt GDAP1 (GDAP1) and mutants TMDscr, HD1scr and HD1del. (B) Distinct mitochondrial localization of mutant proteins in transfected COS-7 cells (a–i). (C) Quantification of mitochondrial morphology. Fission activity was lost by HD1 scrambling or deletion. Bars, 10 µm.

Next, we examined the role of the aa sequences of the TMD and HD1 on mitochondrial targeting and fission activity. To this end, we constructed GDAP1 mutants with scrambled TMD and HD1 (TMDscr, HD1scr) and a mutant lacking HD1 (Fig. 4A; TMD deletion causes loss of mitochondrial targeting and activity; [19]. TMDscr strictly colocalized with mitochondria (Fig. 4B a–c; Suppl. Fig. S4) and displayed full fission activity (Fig. 4C) indicating that the native aa sequence is not critical. Scrambling or deletion of HD1 (Fig. 4A) did not interfere with mitochondrial targeting (Suppl. Fig. S4), but extensive mitochondrial aggregation and tubulation was observed (Fig. 4B d–i). Expression of both of these constructs leads to a significant reduction of cells with fragmented mitochondrial morphology compared to wt GDAP1 (Fig. 4C). Thus, both the presence of HD1 and its correct aa sequence are essential for GDAP1 activity.

MOM targeting of TA proteins has been suggested to depend on short TMDs [6], [25]. GDAP1-variants with different TMD lengths (minus 1, plus 1, 3 or 5 aa) but unchanged mean hydrophobicity are still targeted to the mitochondria (Suppl. Fig. S3). The variant with five additional aa is slightly but significantly mislocalized to the ER. None of these proteins displayed detectable alterations in fission activity. Thus, the TMD length influences the specificity of MOM targeting but as sufficient protein appears to be still targeted to the mitochondria, not GDAP1 fission activity.

### The specific nature of the GDAP1 TMD and its C-terminus is not required for fission activity

**Figure 5:**
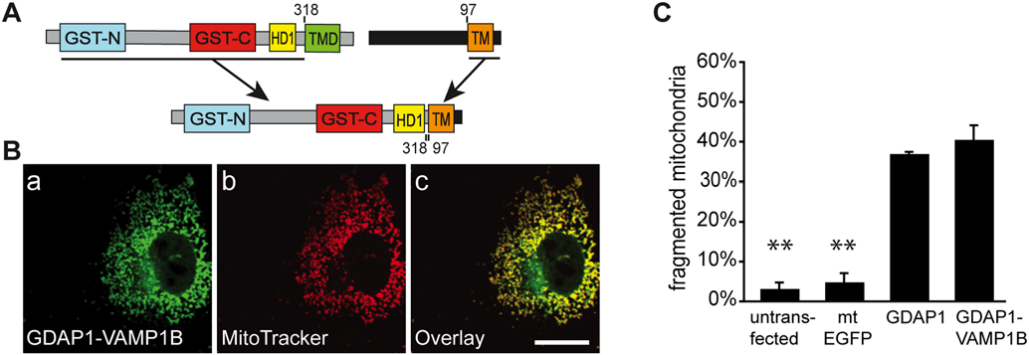
Function of TA in GDAP1 fission activity. (A) Construction of chimera GDAP1-VAMP1B. (B) COS-7 cells transfected with GDAP1-VAMP1B display mitochondrial localization and fragmented mitochondria comparable to full-length GDAP1 (a–c). (C) Quantification reveals no difference in fission activity between GDAP1 and GDAP1-Vamp1B. Bars, 10 µm.

We next asked whether the particular TMD and the flanking GDAP1 C-terminus are critical for fission-inducing activity. Thus, we constructed a chimeric protein in which the TMD and the C-terminus of GDAP1 are replaced by that of VAMP1B, a tail-anchored MOM protein with no known function in mitochondrial fission [25]. The chimeric protein was targeted correctly to mitochondria (Fig. 5B a–c; Suppl. Fig. S4) and retains full fission activity (Fig. 5C). We conclude that there is no functional relevance of the primary sequence of the GDAP1 TMD and its neighboring C-terminus in GDAP1-mediated mitochondrial fission.

### TMD hydrophobicity does not influence GDAP1-induced fission of mitochondria

**Figure 6:**
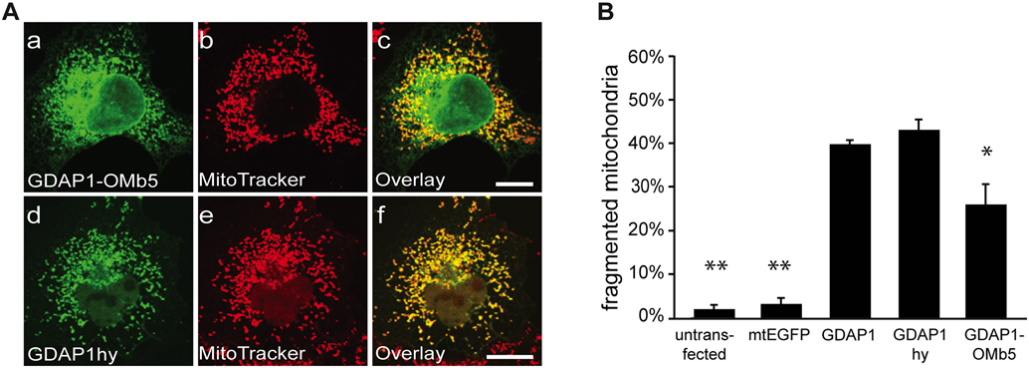
Influence of TMD hydrophobicity on GDAP1 fission activity. (A) COS-7 cells expressing either GDAP1-OMb5 (a–c) or GDAP1hy (d–f) were co-stained with MitoTracker. Both proteins show mitochondrial localization, with some mislocalization of GDAP1-OMb5 to the ER (for quantification, see Suppl. Fig. A3). (B) Quantification of mitochondrial morphology. Bars, 10 µm.

Wattenberg *et al* [26] showed previously, that the hydrophobic/hydrophilic balance is crucial for the targeting of TA proteins. However, so far nothing is known about whether the TA hydrophobicity also influcences the proper function of TA proteins. Hence, we tested whether TMD hydrophobicity has an impact on GDAP1 fission function. Computer analysis revealed that TA mitochondrial dynamics factors can be divided into one group with high TMD hydrophobicity and one with limited hydrophobicity [Table 1; [14]]. Both GDAP1 and VAMP1B contain a strongly hydrophobic TMD. Thus, the previously observed full fission-inducing activity of GDAP1-VAMP1B and TMDscr might be attributed to similar TMD hydrophobicities. To test whether a less hydrophobic TMD would have an effect, we constructed the chimera GDAP1-OMb5 containing the less hydrophobic TA of mitochondrial cytochrome b5 (Table 1). This chimera was only partially targeted to mitochondria (Suppl. Fig. S4), inducing reduced levels of mitochondrial fragmentation (Fig. 6B), with significant proportion of GDAP1-OMb5 directed to the ER (Fig. 6A a–c). Control experiments revealed a comparable ER mislocalization for wt hOMb5 and ratOMb5 in our system rendering this approach suggestive but inconclusive (data not shown). Hence, we generated GDAP1hy, a GDAP1 variant with four TMD aa exchanged for less hydrophobic aa (Table 1). GDAP1hy colocalized exclusively with MitoTracker (Fig. 6A d–f; Suppl. Fig. S4), confirming that a less hydrophobic TMD still retains MOM specificity [14] and full mitochondrial fission activity (Fig. 6B). We conclude that GDAP1 fission activity is not influenced by its TMD hydrophobicity.

## Discussion

GDAP1 is a mitochondrial fission factor of the MOM and mutations affecting this protein cause a subtype of the inherited neuropathy CMT [19]. The knockdown of GDAP1 leads to elongated mitochondria [19]. Overexpression of GDAP1 induces mitochondrial fission in cells that endogenously express GDAP1 (i.e. SH-SY5Y [21]) and in cells with no GDAP1 expression (i.e. HeLa cells or COS7 cells [19]). Mutated forms of GDAP1 found in CMT patients have no or reduced fission activity [19]. In this study, we have analyzed the topology of GDAP1 and its mitochondrial targeting in conjunction with GDAP-mediated mitochondrial fission. Our results define GDAP1 as a classical TA protein that spans the MOM once with its C-terminal TMD. The TMD and its bordering basic aa in the IMS are crucially involved in mitochondrial targeting and membrane insertion. Positively charged aa flanking the TMD on the cytosolic side control both mitochondrial targeting and the fission function of GDAP1. The correct aa sequence of the second hydrophobic and cytosolic HD1 domain is essential for mitochondrial fission mediated by GDAP1. Furthermore, our data show that signals that determine the targeting of GDAP1 to mitochondria as part of the TA are also crucial for the fission function of GDAP1.

**Figure 7:**
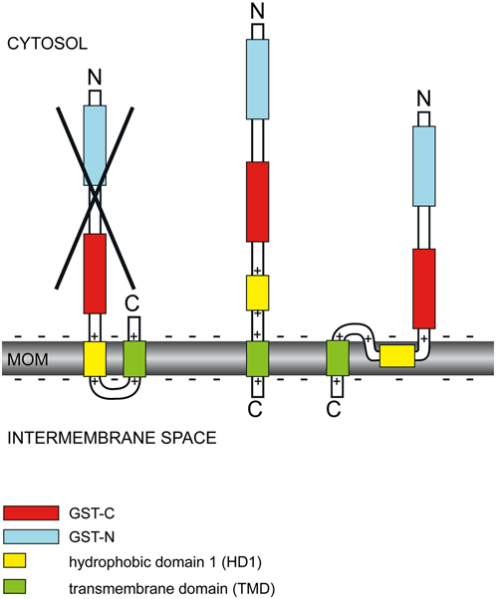
Model of possible GDAP1 membrane topologies. Experimentally confirmed is a single transmembrane span with the C-terminus in the intermembrane space and the N-terminus in the cytosol. Within this topology two different arrangements of the HD1 are represented. Crossed-out: Experimentally disproved topology. MOM, mitochondrial outer membrane.

Concerning mitochondrial targeting, our protease-protection assays revealed that GDAP1 spans the MOM once with its C-terminal TMD. The N-terminal part with the GST domains is located in the cytosol. The short C-terminal tail is located in the IMS. These findings are in agreement with the definition of classical TA-proteins that span the membrane with a single transmembrane domain close to the C-terminus [5]. Our results demonstrate that the HD1 does not span the MOM. Whether the HD1 is located in the cytosol or is embedded within the bilayer plane, cannot be discriminated (modeled in Fig. 7). Furthermore, we show that GDAP1 integrates post-translationally into membranes *in vitro* comparable to the integration of the classical TA protein Fis1. We found that GDAP1 integrates into the membrane exclusively dependent on the TA-domain and not on other cytosolic domains like an N-terminal targeting sequence, the GST-domains, or HD1. In contrast, the two TMD-containing MOM protein Mfn2 did not integrate in our assay system. A similar integration of *in vitro* translated TA-proteins into the MOM has previously been demonstrated in digitonin-permeabilized HeLa cells [17].

The classification of GDAP1 as a TA protein of mitochondria is further supported by the following results: First, MOM TA protein targeting is regulated by basic aa flanking the TMD [5]. In concurrence with this fact, we found that mitochondrial targeting of GDAP1 is critically dependent on clusters of positively charged aa surrounding the TMD. Second, targeting to the MOM is dependent on a short TMD [5]. Indeed, a five aa length extension of the TMD of GDAP1 leads to aberrant targeting of the extended protein to the ER. Third, the TA-domain of GDAP1 and its adjacent C-terminal sequences can be replaced by a heterologous TA-domain and the chimeric protein still maintains mitochondrial targeting. Fourth, like for other TA-proteins, GDAP1-targeting and integration into the membrane is impaired by large C-terminal tags like EGFP [14].

In contrast to the clusters of basic residues surrounding the TMD of GDAP1, the basic residues K291 and R310, adjoining the more N-terminally located HD1, are not required for MOM targeting. They are, however, essential for GDAP1-mediated fission activity. Similarly, HD1 deletion or scrambling of its aa sequence severely impair fission activity of the mutant GDAP1 proteins but without affecting mitochondrial localization. These experiments indicate a key role for HD1 in GDAP1 function without major involvement in mitochondrial targeting and GDAP1 insertion into the MOM. Indeed the R310Q mutation, which has lost the ability to induce mitochondrial fission, was found in CMT patients [19].

Potential GST activity of GDAP1 might modify the lipids of the MOM to allow fission since the GDAP1 sequence harbors both a GST-N and a GST-C domain [27]. This suggestion remains hypothetical, however, since no GST activity has been detected using truncated (GDAP1Δ324–358 and GDAP1Δ334–358) recombinant protein expressed in bacteria [22]. Several mitochondrial dynamic factors are TA-proteins (Table 1) and thus, we reasoned that the GDAP1-TA domain might play a functional role in mitochondrial fission activity. Little is known about the functional relevance of TA domains. In yeast Habib et al. [18] replaced the TA-domain of Fis1 by the TA-domain of TOM5 and TOM6. Both chimeric proteins could restore the mitochondrial morphology phenotype in Δfis1 yeast strains indicating that the TA of Fis1 does not have a functional role. Yet, the Fis1-TOM6 fusion protein was targeted to and stabilized the TOM complex in Δtom6 yeast strains, leaving the possibility that the TA domain of Tom6 has functional relevance [18]. If we apply a very restricted definition, which limits the TA to the TMD, our results reveal no effect of the domain on the fission activity. However, the common definition of the TA-domain includes the TMD and the flanking aa concurrently needed for correct targeting [5]. Intriguingly, our results show that the cluster of basic aa N-terminally bordering the TMD is not only required for mitochondrial localization of GDAP1 but these aa are also of functional importance. Even individual aa of this cluster are concomitantly crucial for GDAP1-induced mitochondrial fission. These findings reveal an overlap of residues belonging to the targeting information of the TA-domain and the fission activity of GDAP1. We conclude that TA-domains in higher eukaryotes can, as shown here for GDAP1, not only serve as a targeting sequence but can also have crucial functional relevance for a mitochondrial dynamics factor.

## Materials and Methods

### Cloning and mutagenesis

The generation of GDAP1-, EGFP-GDAP1- and GDAP1-EGFP- expression constructs was described previously [19]. Vamp1B and OMb5 cDNAs were amplified from cDNA (P. Berger, PSI, Villigen, Switzerland). Point mutations were generated according to the protocol of the QuickChange mutagenesis kit (Stratagene). The chimeric proteins GDAP1-Vamp1B and GDAP1-OMb5, ratOMb5, TMDscr, HD1scr, HD1del, TMD+1V, TMD+3V, TMD+5V, TMD-1V and FLAG-tagged GDAP1 were generated by PCR as described in the online supplemental material (Materials and Methods S1). All cDNAs were cloned into the pGEM-T vector (Promega), subcloned into pcDNA3.1 (Invitrogen) and verified by sequencing. EGFP-C1/N1 vectors, mtDsRED2 and mtEGFP were obtained from Clontech Laboratories, Inc.

### Antibodies

Polyclonal rabbit anti-GDAP1 antibodies have been previously described [19]. The monoclonal mouse anti-FLAG M2 and anti-actin were from Sigma-Aldrich, anti-OPA1 and anti-cytochrome C from BD Biosciences, the monoclonal mouse anti-PDI from Stressgen, anti-GAPDH from HyTest Ltd. and anti-Porin from Calbiochem.

### Cell culture and immunohistochemistry

COS-7 cells and HeLa cells were cultured in DMEM (Invitrogen) containing 10% FCS (Brunschwig) until 80% confluent. COS-7 cells were transfected using Fugene 6 (Roche), HeLa cells using Lipofectamine2000 (Invitrogen) according to the manufacturers' protocols. Cells were fixed at the indicated time points. Immunofluorescence procedures were performed as described previously [19]. To label mitochondria, cells were incubated with MitoTracker Red (Molecular Probes) prior to fixation according to the manufacturer's recommendations. Cells were observed either with a Zeiss Axioplan microscope equipped for epifluorescence and a Zeiss MRM camera or with a confocal inverted microscope (Zeiss LSM 520-NLO) using argon and helium-neon lasers. All images were imported into Photoshop CS (Adobe) for pseudo-coloring, merging, cropping and linear contrast adjustment. To determine the percentage of colocalization of the GDAP1 signal with the mitochondrial marker MitoTracker, we analyzed single plane confocal images using the IMARIS colocalization tool (Bitplane AG). To reduce background signals for the quantitative analysis, the thresholds for the colocalization studies were set at “30” for the green channel and “40” for the red channel as recommended by the software. 85% of the GDAP1 signal colocalized with the MitoTracker signal. For better comparison, this value was set to 100%. The average and the standard deviation of three experiments with 15 pictures per condition were determined, and statistical significance assessed with a two-tailed unpaired *t*-test. For quantification of the mitochondrial morphology, 450 to 600 transfected cells were counted and categorized into five distinct mitochondrial morphologies: Aggregated, tubular, mixed, vesicular and fragmented as previously described [19]. For clarity reason, only the percentage of fragmented mitochondria is shown. Results are shown as average and standard deviation of the percentage of cells with fragmented mitochondrial morphology from three independent transfection experiments. Statistical significance: Two-tailed unpaired t-test. Error bars: Standard deviation; *P<0.05; **P<0.01.

### Subcellular fractionation and protease digests

HeLa cells were transfected with either GDAP1 or GDAP1-FLAG expression constructs. After 24 hours, fractions were isolated and mitochondria were enriched as described previously [19]. Freshly isolated mitochondria in cell fractionation buffer (210 mM mannitol, 70 mM sucrose, 1 mM EDTA, 10 mM Hepes-NaOH pH 7.5) were pooled and processed as described by Olichon, et al. [23]. SDS-PAGE and Western blotting procedures were performed as described [19], chemiluminescence using CDP-Star (Roche) or ECL Western blotting detection reagents (GE Healthcare) was detected with Fuji Medical X-ray films (Fujifilm). Three independent transfections were analyzed for quantification by densitometry.

### In vitro transcription/translation and MOM integration assay

Proteins were synthesized *in vitro* from the respective cDNAs in the pcDNA3.1 vector using the TNT Quick Coupled Transcription/Translation System (1 µg plasmid / reaction). The protocols for membrane integration and differential centrifugation were adapted from Niemann, et al. [19]. The integration assay was performed as follows: Post-nuclear supernatant (800 µg total protein) or the mitochondria-enriched fraction P1 (150 µg total protein) in cell fractionation buffer were incubated with *in vitro*-synthesized proteins for 60 minutes at 4°C. Both subsequent centrifugation steps were performed for 20 minutes at 10000 *g.* Equal volumes of supernatants and pellets were analyzed by autoradiography using ENHANCE (Perkin Elmar) according to the manufacture's protocol. To confirm the integration of *in vitro* translated GDAP1-FLAG this radiolabeled protein was digested with 50 mg/ml proteinase K for 30 min at 4°C without (pure *in vitro* translat) or after membrane integration (60 min incubation with post nuclear supernatant on ice). The digest was stopped with 5 mM PMSF for 10 min. Immunoprecipitation was done with the anti-FLAG antibody (s.a.) in IP-buffer (50 mM Tris pH 7.5, 150 mM NaCl, 0.1% SDS, 1% Triton X-100, 1 mM PMSF, 1∶100 Proteinase Inhibitor Cocktail (Sigma)). The samples were incubated with the antibody at 4°C for 2 h. Protein A-sepharose (GE Healthcare) was added for an additional hour. Samples were washed three times in IP-buffer, boiled at 90°C for 10 min in SDS-buffer (50 mM Tris/HCl pH 6.8, 2% SDS, 10% glycerol) and analyzed by autoradiography.

## Supporting Information

Figure S1 GDAP1-EGFP is peripherally attached to the MOM. (A) COS-7 cells were transiently transfected with either mtEGFP (a–c), wt GDAP1 (d–f), or the C-terminal EGFP-tagged construct GDAP1-EGFP (g–i). Fifteen hours after start of transfection, cells were co-stained with MitoTracker to analyze mitochondrial localization. GDAP1-EGFP shows only partial mitochondrial localization. In addition, the expression of GDAP1-EGFP causes mitochondrial aggregation. (B) Upper panel: Cartoon of a cell before and after permeabilization of the cell membrane with low concentrations of digitonin showing the release of cytosolic proteins (a, a′). Subsequent treatment with trypsin digests cytosol-exposed membrane-bound protein parts (a″; yellow dots, cytosolic proteins; blue dots, cytosolic parts of MOM-attached proteins; green dots, proteins of the intermembrane space; red dots, proteins of the matrix). The used digitonin and trypsin concentrations do not affect the mitochondrial membrane integrity (Lorenz et al., Nat. Methods 3, 205–210, 2006). Lower panel (b–g): GDAP1-EGFP and mtDsRED expressing COS-7 cells were permeabilized with 50 µM digitonin and treated in parallel with 250 µM trypsin for the indicated time points. Images were taken after permeabilization and trypsin digest. The cytosolic GDAP1-EGFP signal is washed out early due to permeabilization of the cell (c). Only the GDAP1-EGFP associated with mitochondria remains detectable but is lost over time of the protease digest (b–d). The mitochondrial targeted marker mtDsRED is not washed out or degraded by the protease (e–g). These results indicate that GDAP1-EGFP partly associates with mitochondria but the long-extended C-terminus fails to translocate across the MOM. Bars, 10 µm.
(10.01 MB TIF)

Figure S2 In-vitro translated GDAP1-EGFP peripherally attaches to membranes. The post-nuclear supernatant of HeLa cells was incubated with the in vitro-translated GDAP1-GFP and the mitochondrial pellet was resuspended in buffer (control), in 1 M NaCl, 0.1 M carbonate (pH 11), or in buffer with 0.1% TritonX-100, and centrifuged to separate the soluble protein supernatants (S) from membranous pellets (P). Upon treatment with sodium chloride or carbonate GDAP1-GFP was extracted as was the intermembrane space protein Cytochrome C [15], [21], whereas the MOM integral protein porin remained in the membrane pellets.
(1.47 MB TIF)

Figure S3 Effect of TMD length on mitochondrial targeting and fission activity. (A) Schema of GDAP1 TMD aa sequence and constructs with altered TMD length. TMD hydrophobicities are given in brackets. (B) Confocal immunofluorescence analysis of transfected COS-7 cells reveals mitochondrial targeting for all recombinant proteins (a–l), albeit for TMD+5 with reduced efficiency (g–i). (C) Quantification of mitochondrial localization. Significant mislocalization was detected for TMD+5. (D) Analysis of fragmentation-inducing activity of mutants revealed no significant difference compared to wt GDAP1 (GDAP1). Bars, 10 µm.
(9.88 MB TIF)

Figure S4 Quantitative analysis of mitochondrial targeting. Quantification of mitochondrial localization of recombinant proteins used in this study in transiently transfected COS-7 cells. Abbreviations are explained in the text.
(2.66 MB TIF)

Figure S5 Quantitative analysis of expression levels. (A) Expression levels of tested GDAP1 point mutants, (B) mutants with varying TMD length, and (C) tagged and chimeric variants of GDAP1 were comparable to wt GDAP1 (GDAP1) protein in transfected COS-7 cells. The abbreviations are explained in the text. Quantification was performed by calculating the ratio of anti-GDAP1/anti-beta actin signals on Western blots of cell lysates from sister plates of those used for morphological analysis (n = 3).
(9.89 MB TIF)

Materials and Methods S1 Supplemental Materials and Methods
(0.05 MB DOC)
