# Supplementary figures and images for: Targeting and Function of the Mitochondrial Fission Factor GDAP1 Are Dependent on Its Tail-Anchor
Source: PLoS One. 2009 Apr 2;4(4):e5160. doi: 10.1371/journal.pone.0005160 (PMC2659752; doi:10.1371/journal.pone.0005160)

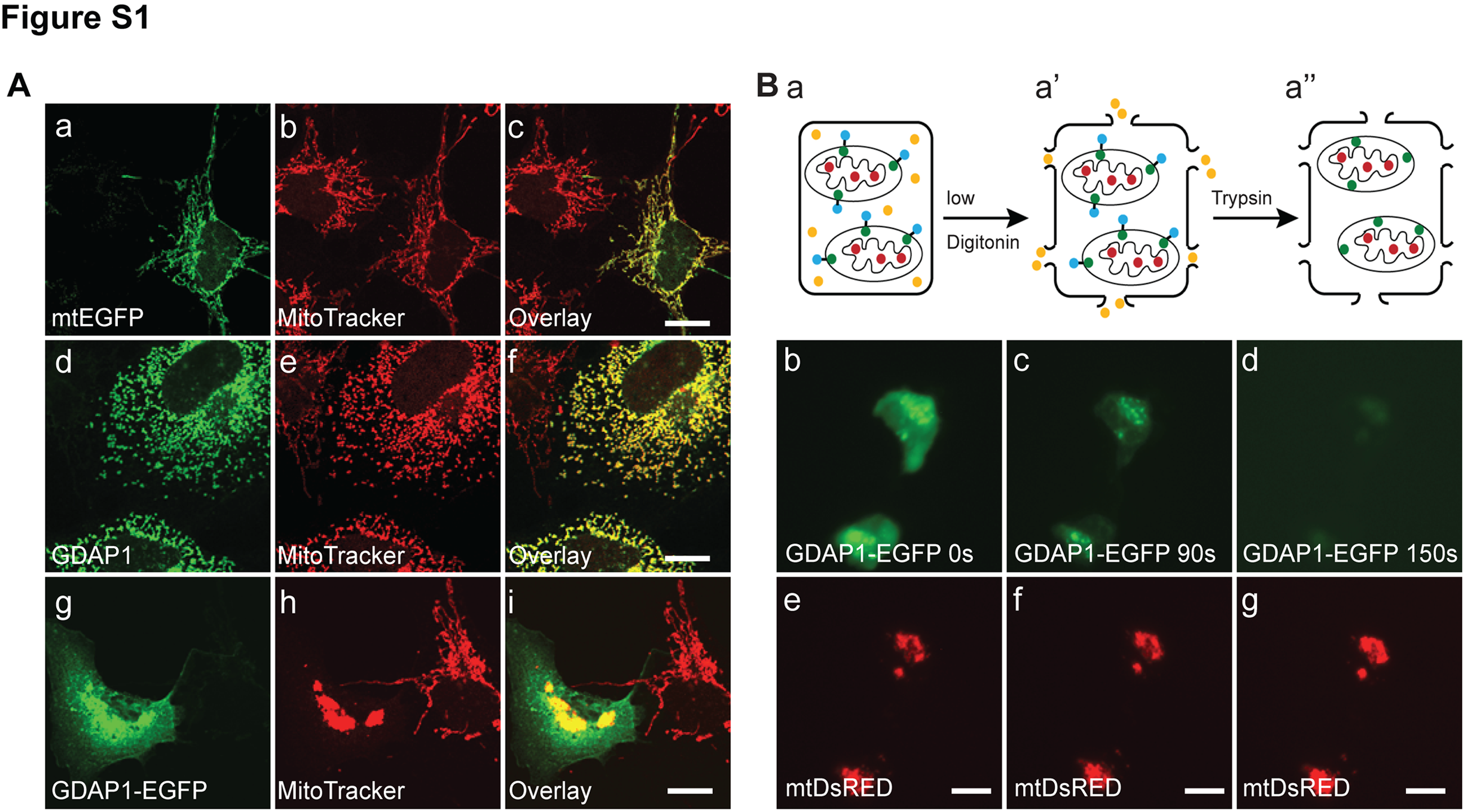

Supplement: Figure S1 — GDAP1-EGFP is peripherally attached to the MOM. (A) COS-7 cells were transiently transfected with either mtEGFP (a–c), wt GDAP1 (d–f), or the C-terminal EGFP-tagged construct GDAP1-EGFP (g–i). Fifteen hours after start of transfection, cells were co-stained with MitoTracker to analyze mitochondrial localization. GDAP1-EGFP shows only partial mitochondrial localization. In addition, the expression of GDAP1-EGFP causes mitochondrial aggregation. (B) Upper panel: Cartoon of a cell before and after permeabilization of the cell membrane with low concentrations of digitonin showing the release of cytosolic proteins (a, a′). Subsequent treatment with trypsin digests cytosol-exposed membrane-bound protein parts (a″; yellow dots, cytosolic proteins; blue dots, cytosolic parts of MOM-attached proteins; green dots, proteins of the intermembrane space; red dots, proteins of the matrix). The used digitonin and trypsin concentrations do not affect the mitochondrial membrane integrity (Lorenz et al., Nat. Methods 3, 205–210, 2006). Lower panel (b–g): GDAP1-EGFP and mtDsRED expressing COS-7 cells were permeabilized with 50 µM digitonin and treated in parallel with 250 µM trypsin for the indicated time points. Images were taken after permeabilization and trypsin digest. The cytosolic GDAP1-EGFP signal is washed out early due to permeabilization of the cell (c). Only the GDAP1-EGFP associated with mitochondria remains detectable but is lost over time of the protease digest (b–d). The mitochondrial targeted marker mtDsRED is not washed out or degraded by the protease (e–g). These results indicate that GDAP1-EGFP partly associates with mitochondria but the long-extended C-terminus fails to translocate across the MOM. Bars, 10 µm. (10.01 MB TIF) [file pone.0005160.s001.tif]

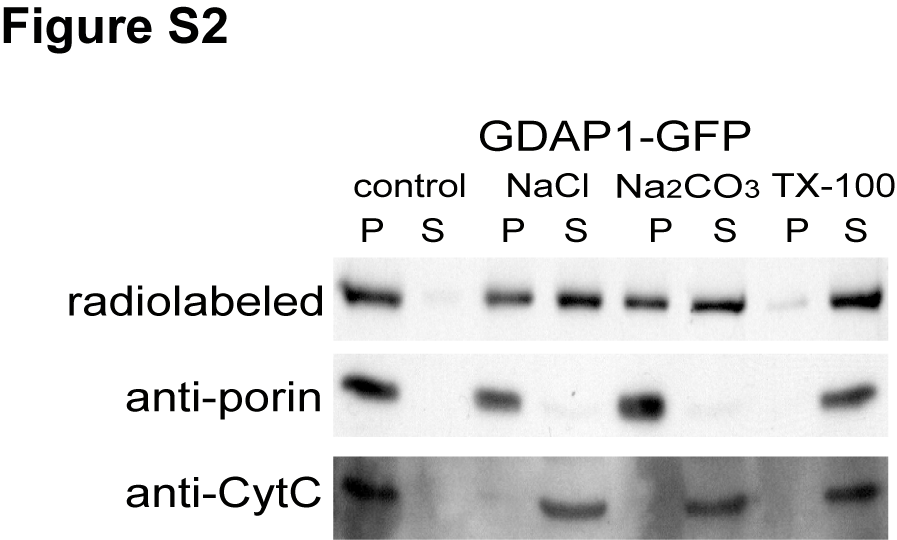

Supplement: Figure S2 — In-vitro translated GDAP1-EGFP peripherally attaches to membranes. The post-nuclear supernatant of HeLa cells was incubated with the in vitro-translated GDAP1-GFP and the mitochondrial pellet was resuspended in buffer (control), in 1 M NaCl, 0.1 M carbonate (pH 11), or in buffer with 0.1% TritonX-100, and centrifuged to separate the soluble protein supernatants (S) from membranous pellets (P). Upon treatment with sodium chloride or carbonate GDAP1-GFP was extracted as was the intermembrane space protein Cytochrome C [15], [21], whereas the MOM integral protein porin remained in the membrane pellets. (1.47 MB TIF) [file pone.0005160.s002.tif]

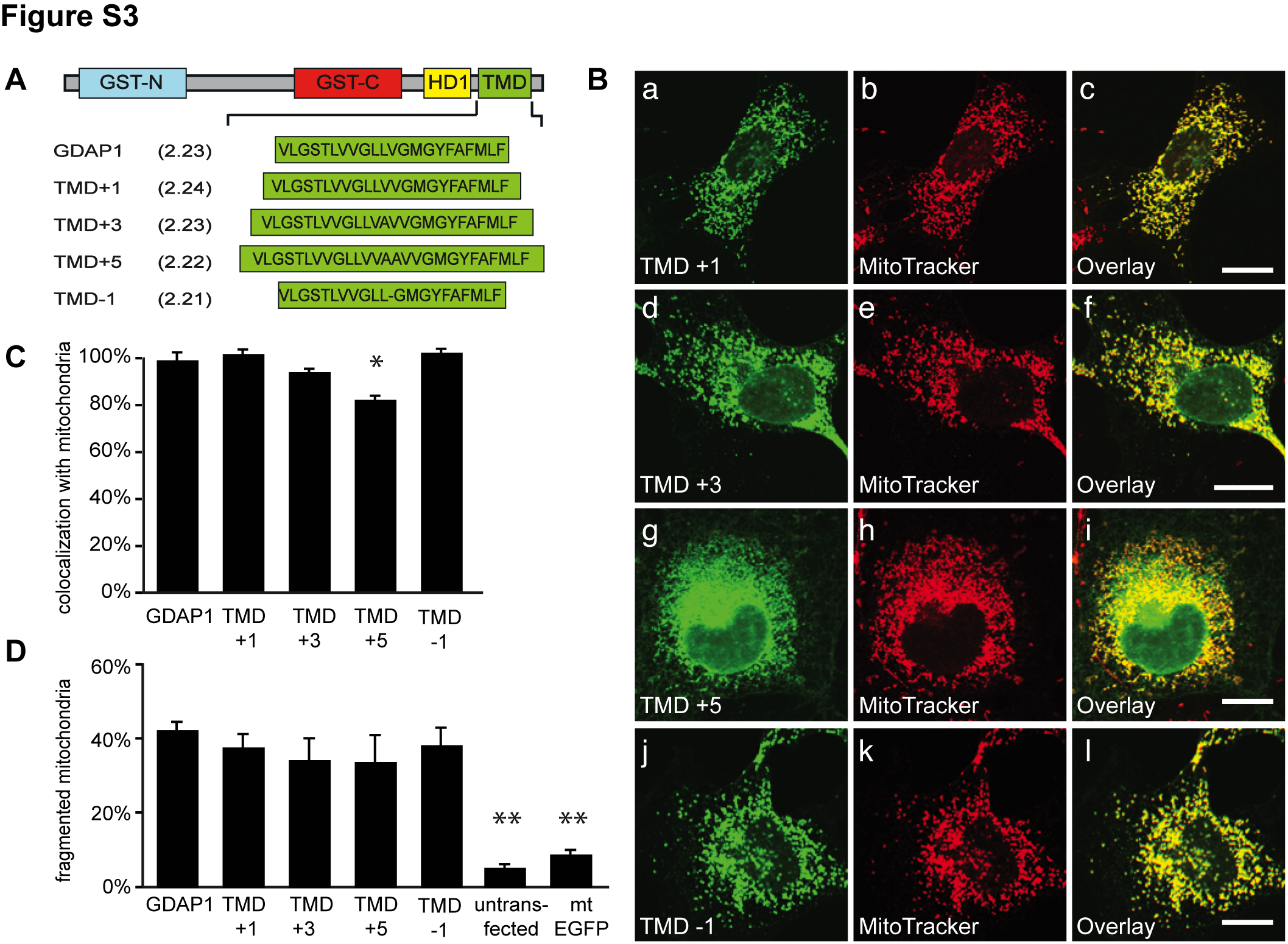

Supplement: Figure S3 — Effect of TMD length on mitochondrial targeting and fission activity. (A) Schema of GDAP1 TMD aa sequence and constructs with altered TMD length. TMD hydrophobicities are given in brackets. (B) Confocal immunofluorescence analysis of transfected COS-7 cells reveals mitochondrial targeting for all recombinant proteins (a–l), albeit for TMD+5 with reduced efficiency (g–i). (C) Quantification of mitochondrial localization. Significant mislocalization was detected for TMD+5. (D) Analysis of fragmentation-inducing activity of mutants revealed no significant difference compared to wt GDAP1 (GDAP1). Bars, 10 µm. (9.88 MB TIF) [file pone.0005160.s003.tif]

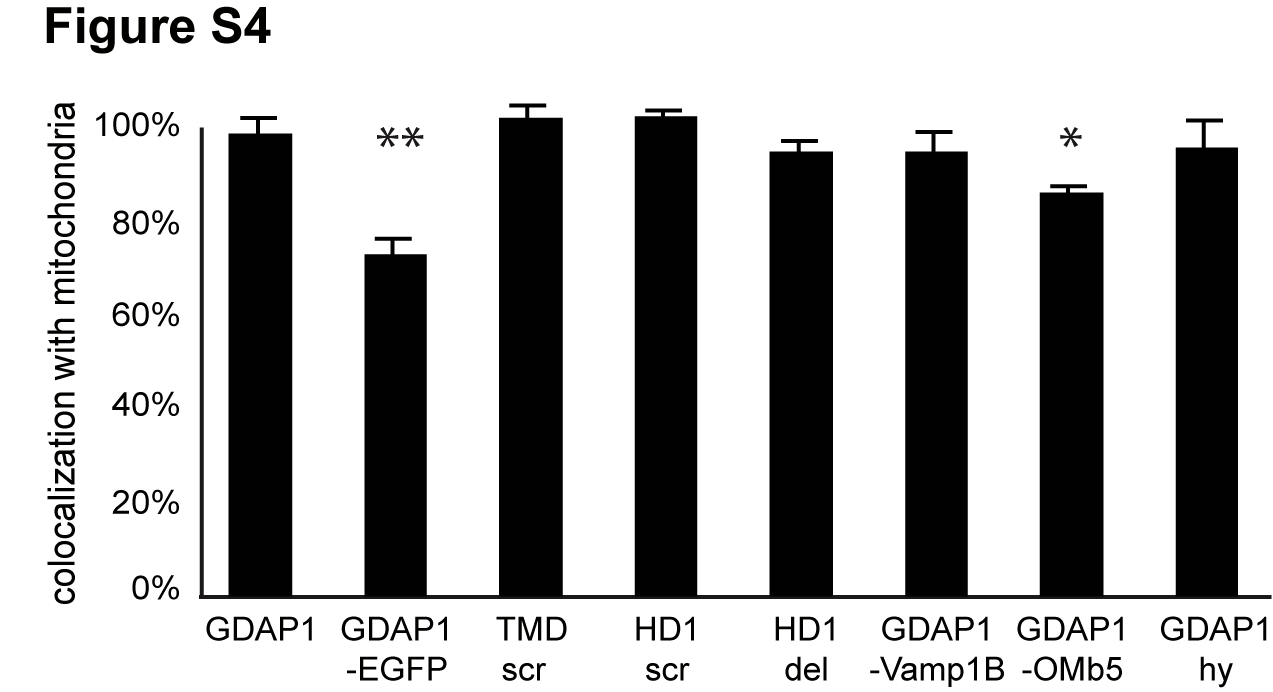

Supplement: Figure S4 — Quantitative analysis of mitochondrial targeting. Quantification of mitochondrial localization of recombinant proteins used in this study in transiently transfected COS-7 cells. Abbreviations are explained in the text. (2.66 MB TIF) [file pone.0005160.s004.tif]

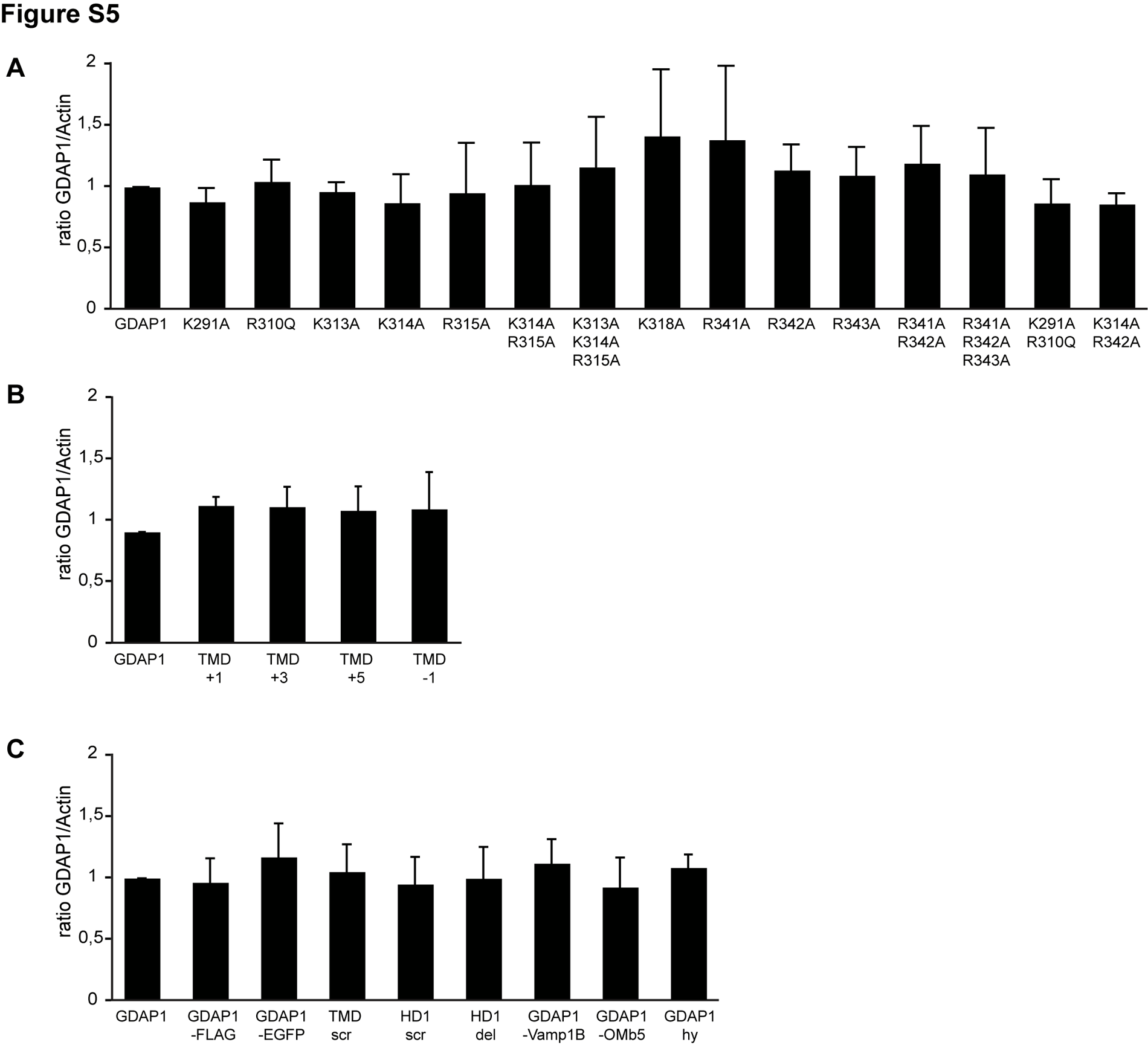

Supplement: Figure S5 — Quantitative analysis of expression levels. (A) Expression levels of tested GDAP1 point mutants, (B) mutants with varying TMD length, and (C) tagged and chimeric variants of GDAP1 were comparable to wt GDAP1 (GDAP1) protein in transfected COS-7 cells. The abbreviations are explained in the text. Quantification was performed by calculating the ratio of anti-GDAP1/anti-beta actin signals on Western blots of cell lysates from sister plates of those used for morphological analysis (n = 3). (9.89 MB TIF) [file pone.0005160.s005.tif]
