# Supplementary material for: Targeting and Function of the Mitochondrial Fission Factor GDAP1 Are Dependent on Its Tail-Anchor
Source: PLoS One. 2009 Apr 2;4(4):e5160. doi: 10.1371/journal.pone.0005160 (PMC2659752; doi:10.1371/journal.pone.0005160)
Supplement: Materials and Methods S1 — Supplemental Materials and Methods (0.05 MB DOC) [file pone.0005160.s006.doc]

# SUPPLEMENTAL MATERIALS AND METHODS

Fluorescence protease protection assay - COS-7 cells were grown on 12-well plates, transfected, and analyzed 24 h later. Cells were washed twice in KHM buffer (110 mM potassium acetate, 20 mM HEPES, 2 mM MgCl_2_) at room temperature (Lorenz et al., 2006), incubated with 50 μM digitonin (Sigma-Aldrich) and 250 μM trypsin (Fluka) in KHM buffer. Images were taken at indicated time points using a Zeiss Observer microscope with a Zeiss MRM camera and AxioVision 4 software, and processed by Adobe Photoshop.

Cloning and site directed mutagenesis - The cDNAs for the various constructs and fusion proteins were constructed as follows:

**GDAP1-FLAG:** PCR amplification on the GDAP1/pGEM-T template using the upper primer 5’-cggatccatggctcggaggcaggac-3’ and the lower primer 5’-ctacttgtcgtcgtccttgtagtctctgggtctaagtgctaa-3’.

**Vamp1B:** PCR amplification from mouse brain cDNA using the upper primer 5’-ctccggagaggaacagacc-3’ and the lower primer 5’-ctgcattaggcaaggaggag-3’.

**GDAP1-Vamp1B:** *Step 1* PCR amplification on the GDAP1/pGEM-T and Vamp1B/pGEM-T templates using the upper primer GDAP1 FP 5’- cggatccatggctcggaggcaggac-3’ and Vamp1B FP 5’-caaaaaaagggccccaaaaatgatgatcatgctgggag-3’ and the lower primer GDAP1 RP1 5’-ctcccagcatgatcatcatttttggggccctttttttg-3’ and Vamp1B RP 5’-ctgcattaggcaaggaggag-3’. *Step 2* PCR amplification on the in Step 1 with overlapping ends generated GDAP1 and Vamp1B PCR products using the primer pair GDAP1 FP and Vamp1B RP.

**OMb5:** PCR amplification from HeLa cell cDNA using the upper primer 5’-ggtggagaggcagtatgtcc-3’ and the lower primer hOMb5 RP1 5’-ggatgcactttctaacttcagca-3’.

**FLAG-hOMb5:** PCR amplification on the hOMb5/p-GEM-T template using the upper primer 5’-taggatccatggactacaaggatgacgatgacaaggcgactgcggaagctagcggc-3’ and the lower primer hOMb5 RP1 (see hOMb5).

**GDAP1-hOMb5:** *Step 1* PCR amplification on the GDAP1/p-GEM-T and hOMb5/p-GEM-T templates using the upper primer GDAP1 FP (see GDAP1-Vamp1B) and hOMb5 FP 5’- caaaaaaagggccccaaaaagttgctgggcatattgg-3’ and the lower primer GDAP1 RP2 5’-ccaatatgcccagcaactttttggggccctttttttg-3’ and hOMb5 RP2 ggatgcactttctaacttcagca-3’. *Step 2* PCR amplification on the in Step 1 with overlapping ends generated GDAP1 and hOMb5 PCR products using the primer pair GDAP1 FP and hOMb5 RP.

**GDAP1-ratOMb5:** PCR amplification on the GDAP1-hOMb5/pGEM-T template using the upper primer GDAP1 FP (see GDAP1-Vamp1B) and the consecutively used lower primer 5’-cacgatggggacaatccaatatgcccattttggggccctttttttggccac-3’, 5’-gacgatacaggaaacctataagaatagcacccacgatggg gacaatccaat-3’ and 5’-gtcgactcaggaggatttgctgtcagcccagaagtgacgatacaggaaacctataag-3’.

**TMDscr:** *Step 1* PCR amplification on the GDAP1/pGEM-T template using the upper primer GDAP1 FP (see GDAP1-Vamp1B) and 5’-cttgtgggacttttggtgagaaggagacttggcagcatg-3’ and the lower primer 5’-cacaaaaagtgccatgccaacttttggggccctttttttggc-3’ and GDAP1 RP3 5’-gtcgactcagaaataatttggtctgggtct-3’. This step produces a C-terminal and a N-terminal GDAP1 fragment excluding the TMD sequence. *Step 2* Elongation of the PCR products from step 1 using the upper primer GDAP1 FP and the lower primer 5’-ccaaaatagctcataccggtcacaaaaagtgccatgccaac-3’ for the C-terminal fragment; the upper primer 5’-atgagctattttggacttcttgtgggacttttggtgagaagg-3’ and the lower primer GDAP1 RP3 for the N-terminal fragment. *Step 3* PCR amplification of the pooled products of step 2 with the upper primer GDAP1 FP and GDAP1 RP3.

**HD1scr*:*** *Step 1* PCR amplification on the GDAP1/pGEM-T template using the upper primer GDAP1 FP (see GDAP1-Vamp1B) and 5’- ctccgatcgctggcactaaatgtgtagaattgaaaacgatc-3’ and the lower primer 5’- gtgtagaattgaaaacgatcttgttaaatgtttttc-3’ and GDAP1 RP3 5’-gtcgactcagaaataatttggtctgggtct-3’. This step produces a C-terminal and a N-terminal GDAP1 fragment excluding the HD1 sequence. *Step 2* Elongation of the PCR products from step 1 using the upper primer GDAP1 FP and the lower primer 5’- gatcggagcggtgttattaaaccgggtggccaaaaaaagggc-3’ for the C-terminal fragment; the upper primer 5’-caattctacacatttagtgccagcgatcggagcggtgttattaaacc-3’ and the lower primer GDAP1 RP3 for the N-terminal fragment. *Step 3* PCR amplification of the pooled products of step 2 with the upper primer GDAP1 FP and GDAP1 RP3.
